# SMART: On-Site Rapid Detection of Nucleic Acid from Plants, Animals, and Microorganisms in under 25 Minutes

**DOI:** 10.3390/bios13010082

**Published:** 2023-01-03

**Authors:** Jun-Yuan Ma, Xiao-Fu Wang, Cheng Peng, Xiao-Yun Chen, Xiao-Li Xu, Wei Wei, Lei Yang, Jian Cai, Jun-Feng Xu

**Affiliations:** 1 State Key Laboratory for Managing Biotic and Chemical Threats to the Quality and Safety of Agro-Products, Zhejiang Academy of Agricultural Sciences, Hangzhou 310021, China; 2 College of Biological and Food Engineering, Fuyang Normal University, Fuyang 236041, China; 3 Key Laboratory of Traceability for Agricultural Genetically Modified Organisms, Ministry of Agriculture and Rural Affairs, Hangzhou 310021, China

**Keywords:** SMART, on-site detection, ultrafast DNA adsorption, RPA

## Abstract

The rapid on-site nucleic acid detection method is urgently required in many fields. In this study, we report a portable and highly integrated device for DNA detection that combines ultrafast DNA adsorption and rapid DNA amplification. The device, known as silicon film mediated recombinase polymerase amplification (RPA) for nucleic acid detection (SMART), can detect target DNA in less than 25 min from plants, animals, and microbes. Utilizing SMART, transgenic maize was rapidly detected with high selectivity and sensitivity. The sensitivity threshold of the SMART for transgenic maize genomic DNA was 50 copies. The detection results of genuine samples containing plants, animals, and microbes by SMART were consistent with the conventional polymerase chain reaction (PCR) method, demonstrating the high robustness of SMART. Additionally, SMART does not require expensive equipment and is fast, affordable, and user-friendly, making it suited for the broad-scale on-site detection of nucleic acids.

## 1. Introduction

As an essential component of life science, nucleic acid testing is widely employed to detect DNA from plants, animals, and microorganisms. Nucleic acid detection is extensively used in disease prevention, food safety, and other fields. Currently, the conventional nucleic acid detection method involves extracting nucleic acid from biological samples and then detecting it by polymerase chain reaction (PCR). PCR is widely used in clinical medicine, agriculture, food safety, and other fields because of its high sensitivity and specificity [1,2,3]. However, PCR requires a skilled operation, complex equipment, multiple enzymes, and heating and cooling cycles [4]. Additionally, PCR is not suited for rapid on-site testing due to its large volume, lengthy detection time, and high cost [5]. Isothermal amplification technology was designed to tackle numerous challenges in PCR detection [6]. Isothermal amplification technology is widely used for rapid on-site detection due to its low cost, speed, and high sensitivity [7]. Loop-mediated isothermal amplification (LAMP) and recombinase polymerase amplification (RPA) are two significant isothermal amplification technologies [8]. The detection process avoids the heating and cooling steps in conventional PCR detection, and nucleic acid amplification can be accomplished using simple equipment with a constant temperature [9]. Moreover, isothermal amplification requires less time, as the detection time of LAMP is only one hour and that of RPA is only 25 min [10,11]. Since the introduction of RPA detection kits, they have been utilized in various disciplines of life science and food safety.

On-site rapid detection is an important avenue of research in gene detection. Currently, numerous studies are attempting to develop swift procedures to facilitate simpler and faster DNA detection. An optimized visual LAMP method was reported that can detect exogenous DNA targets from two genetically modified (GM) soybean samples within 40 min [11,12]. Despite a significant reduction in detection time, the method does not eliminate the extraction and purification steps in field detection. Some studies have integrated DNA extraction and amplification onto microfluidic chips based on LAMP to simplify the operation steps of on-site detection; the whole process might be performed automatically by optimizing microstructures and controlling the rotational speed of the chip [13]. However, the microfluidic chip has a complex structure, and the amplification of target DNA requires a thermostatic amplifier, limiting its use for on-site detection. Liu et al. designed simple paper microfluidics for rapid on-site detection [14]. Although extracted DNA on filter paper could be directly detected without elution, the method required amplicons detection equipment. A new nucleic acid detection method has been reported to develop a simple and low-cost DNA detection system. By simplifying DNA extraction steps and combining RPA with flow test paper, rapid and highly sensitive detection of genetically modified crop molecules (*Cauliflower mosaic virus 35S promoter* [35S] *and Agrobacterium tumefaciens NOS terminator* [NOS]) has been made possible [15]. Although the steps of on-site detection have been optimized, the method still requires laboratory instruments and commercial extraction kits. Some studies proposed a portable nucleic acid detection method based on 3D printing that achieves isothermal amplification of target genes using a water bath, eliminating the need for an isothermal amplification instrument [16]. However, this method has a wide temperature range for the water bath, which impacts the detection of results.

In summary, these methods can be applied to rapid on-site detection, but they simplify some links in nucleic acid detection. They do not achieve the integration of extraction, amplification, and detection, thus failing to meet the requirements of rapid on-site detection. Therefore, developing a biosensor that integrates nucleic acid extraction, amplification, and detection is crucial to achieve high sensitivity, specificity, and rapid sample detection [17].

In this study, we present the development of a portable, highly integrated SMART device for ultrafast DNA adsorption and rapid DNA amplification. In this device, the target DNA is rapidly adsorbed by the silicon membrane (Figure S1). It is exponentially amplified by RPA to rapidly detect DNA from plants, animals, and microorganisms within 25 min. SMART can detect transgenic maize DNA with high sensitivity and specificity. Due to its simple structure and cost-effectiveness, SMART can remedy conventional PCR detection’s shortcomings and potentially become an analytical platform.

## 2. Materials and Methods

### 2.1. Materials

In this study, 3 kinds of samples were selected, including 7 samples of plants (non-GM seeds of soybeans, rapeseed, and rice, seeds of GM maize DR12-5, GM rapeseed MS1 × RF1, GM soybean 356043, and GM corn NK603), 4 samples of animals (chicken, beef, pork, and duck), and 4 samples of microorganisms (*Enterococcus*, *Salmonella*, *Escherichia coli*, and *bacterial fruit of blotch* (Ac)). Non-GM materials (plant), chicken, beef, and pork (animal), *Enterococcus*, *Salmonella*, and *Escherichia coli* (microorganism) were employed for the experiment of ultrafast adsorption of DNA from different samples. GM materials were chosen for the experiment to verify the sensitivity and specificity of SMART. GM maize DR12-5, duck, and Ac were selected for on-site detection with SMART. These samples were obtained from our laboratory (Hangzhou, China). All of the primers and probes were synthesized by the Sangon Biotech company (Shanghai, China). Detailed information on all primers and probes is provided in Table S1.

### 2.2. Design and Manufacturing of SMART

**Figure 1:**
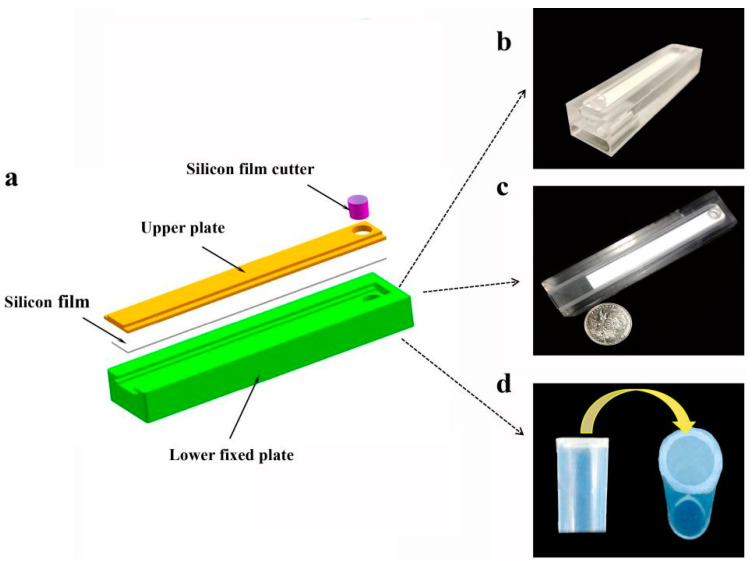
Structure and appearance of SMART: (**a**) the exploded view of SMART; (**b**) side view of SMART; the round hole is for installing the temperature sensor, and the square groove is for the heating plate; (**c**) the image of SMART (the coin’s diameter is 25 mm); (**d**) the image of the silicon film cutter.

The structure and components of SMART can be seen in Figure 1 and were designed using SOLIDWORKS^®^2022. The SMART primarily comprised a silicon film cutter, an upper plate, a silicon film, and a lower fixed plate. The overall dimensions of the polymethylmethacrylate (PMMA) plate were 100 mm in length, 20 mm in width, and 15 mm in height. The exploded view of SMART is depicted in Figure 1a, and the actual shooting image of SMART is shown in Figure 1c. The upper plate of SMART was used to fix the silicon film and silicon film cutter, and it prevented the extracting solution from evaporating. The bottom of the lower fixed plate had a 5 mm diameter isothermal amplification groove, which was used to target gene amplification. One of the circular holes on the right side of the lower fixed plate was used to install a 4.5 mm diameter cylindrical temperature sensor (Figure 1b). The length and width of the square groove on the right side of the lower fixed plate, which was used to install a heating sheet, were 18 mm and 4 mm, respectively (Figure 1b). To achieve directional cutting of DNA-adsorbed silicon film, a silicon film cutter consisting primarily of polyethylene was designed. A polyethylene sealing film and a cutting body comprised the silicon film cutter. Images of the silicon film cutter are shown in Figure 1d. Without the completion of the cutting step, the silicon film cutter might slide with the upper plate.

### 2.3. The Operation of SMART

**Figure 2:**
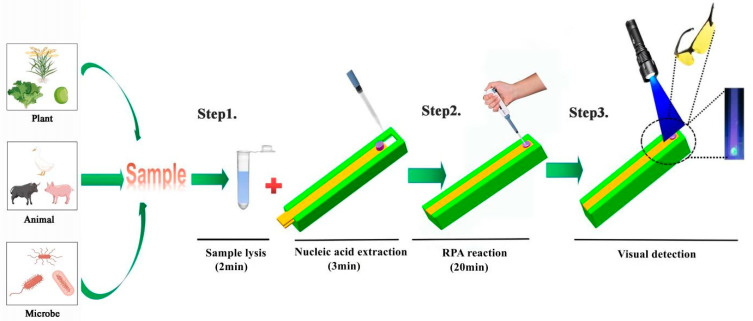
Operation process of SMART: **Step 1** sample lysis and ultrafast DNA adsorption; **Step 2** rapid amplification of target genes; **Step 3** visual detection.

The operational process of the SMART is shown in Figure 2. The detailed operating process is depicted in Figure S2. SMART is capable of ultrafast DNA adsorption from plant, animal, and microbial sources. The ultrafast adsorption of sample DNA has three steps: sample lysis, sample DNA ultrafast adsorption, and sample DNA purification. For ultrafast adsorption of nucleic acids, the sample with a corresponding amount was transferred to a 2.0 mL tube. Then, 200 µL of lysis liquid was added to the tube to resuspend the sample, and the tube was vigorously shaken. After one minute of incubation, the upper plate of the SMART was opened. The 60 µL lysed mixture was added to the silicon film on the SMART, and the upper plate was closed. After two minutes of incubation, the upper plate of the SMART was opened. To remove impurities from the lysis solution, 200 µL wash liquid containing 60% ethanol was injected into the silicon film on the SMART (incubated for 2 min). Step 1 of Figure 2 depicts the ultrafast adsorption steps of the DNA sample.

The silicon film on the upper plate was sliced using the silicon film cutter. Then, from the top of the silicon film cutter, 50 µL of the RPA reaction solution was added to the silicon film that had been cut. Additionally, the upper part of the silicon film cutter was sealed with a polyethylene sealing plate film. The temperature sensor and heating block were inserted into the circular hole and square groove on the right side of the lower fixed plate, respectively, to amplify the target DNA. The power was switched on to amplify the target DNA. The RPA reaction was incubated at 38 °C for 20 min. Figure 2 depicts step 2 of the amplification steps of the DNA sample.

The visual green fluorescence result for product detection was obtained with glasses under a hand-held lamp (LUYOR-3415RG). Figure 2 depicts step 3 of visual detection steps of the DNA sample.

### 2.4. Establishment of SMART Ultra-Fast DNA Adsorption System

#### 2.4.1. Screening of Adsorption Materials

In this work, four adsorption materials were used as analytical samples: Waterman medium-speed qualitative filter paper (Hangzhou, China), 0.58 mm silicon film (Hangzhou, China), cellulose acetate film (Chengdu, China), and A4 paper (Jiangsu, China) with a weight of 70 g and a thickness of 0.1 mm. 

Firstly, the four adsorption materials were cut into 90 mm × 9 mm rectangles. Next, we used 200 µL of 3M guanidine hydrochloride (GuHCl) lysis buffer to lyse the maize seeds (non-GM), using 4 adsorption materials to adsorb the DNA of the sample quickly, and 200 µL wash liquid containing 80% ethanol to remove impurities from the film. Moreover, discs (1 mm in diameter of adsorption material) from different materials were dug with a disposable punch (Figure S3). Discs were then used as templates for RT-PCR amplification of the endogenous plant gene (18S rRNA). Each experiment in this study was repeated three times independently. The evaluation method was used for subsequent molecular tests, such as the screening of lysis buffer and washing buffer. Meanwhile, the pore size and fiber structure of the four adsorption materials was scanned by electron microscopy (HITACHI, Tokyo, Japan).

#### 2.4.2. Screening of Lysis Buffer

This study used the lysis buffer containing 10% CTAB, 20% SDS, 1M-NaOH, and 4M-GuHCl to resuspend samples and the adsorption material to adsorb DNA which served as a template to conduct the PCR reaction using the Real-Time PCR System (CFX96, Bio-Rad, Hercules, CA, USA).

#### 2.4.3. Screening of Washing Solution

A total of 5 different concentrations of wash buffer were considered for removing contaminants: 20, 40, 60, 80, and 100% ethanol. Using the adsorption material to adsorb DNA from maize seeds (non-GM), which was used as a template to amplify the endogenous plant gene (18S rRNA) via the RT-PCR to evaluate the washing performance of these wash buffers. 

#### 2.4.4. Ultrafast Adsorption of DNA from Different Samples

SMART was used to adsorb DNA from the plant (non-GM soybean seeds, non-GM rape seeds, and non-GM rice seeds), animal (chicken meat, beef, and pork), and microbial (*Enterococcus*, *Escherichia coli*, and *Salmonella*) samples, to determine its feasibility. Similar extraction was also performed as a comparative test using a commercial DNA extraction kit (Tiangen, Beijing, China). 

There were differences in the pretreatment of various samples (before the DNA was adsorbed). For the plant samples, fresh plant leaves were crushed using a disposable grinding pestle for the plant samples. Then, leaf powder (30 mg) was transferred to a 2 mL tube. The seeds were smashed to powder by a hammer, and seed powder (30 mg) was transferred to a 2 mL tube. For the animal samples, the meat was crushed using a disposable plastic pestle, and the crushed sample (30 mg) was transferred into a 2 mL tube. For the microbial samples, 40 µL of cultured bacterial strains (2.0 × 10^2^ CFU/µL) was transferred into a 2 mL tube. The DNA was then extracted using the SMART ultra-fast DNA adsorption methodology. The genomic DNA acquired by the adsorbing material and the commercial DNA extraction kit was used as a template to amplify the endogenous gene from plants, animals, and microbes (endogenous genes of plants and animals were 18S rRNA, and endogenous gene of microbes was 16S rRNA) using RT-PCR.

### 2.5. DNA Amplification Reactions and Product Detection

RPA reactions were performed according to the manufacturer’s instructions (Exo kit/basic kit, Amp-future Biotech, Weifang, China). All reactions contained 29.5 µL buffer A, 2.5 µL buffer B, 1 pellet of the lyophilized enzyme, 2 µL forward and reverse primers, 0.6 µL probe, 2 µL DNA templates (the addition or absence of templates in the RPA reaction system depended on different experiments), and 11.5 µL nuclease-free water. The RPA reaction was performed at temperatures ranging from 34 to 42 °C for 20 min.

The PCR reaction mixture (volume: 20 µL), contained 8 µL 2 × TaqMan Universal Master mix (Applied Biosystems, Foster City, CA, USA), 8 µL nuclease-free water, 0.8 µL forward and reverse primers, 0.4 µL probe, and 2 µL DNA template (or 1 mm size of membrane). PCR reactions were performed on the CFX96 real-time PCR system. The PCR reaction program included an initial denaturation at 95 °C for 5 min, followed by 40 cycles of denaturation at 95 °C for 10 s, and annealing at 58 °C for 60 s, with a final extension at 72 °C for 7 min. The threshold time (Tt) reflects the minimum time required to produce a detectable fluorescence signal. Moreover, the visual green fluorescence results for RPA product detection on the silicon film were obtained with glasses under a hand-held lamp (LUYOR-3415RG).

### 2.6. Temperature Control Experiment

The RPA reaction was performed at different temperatures (34, 36, 38, 40, and 42 °C) to evaluate the efficiency of SMART temperature control. Similar work was also performed using a thermal block to compare the result. The genomic DNA (800 copies) of the GM maize double-resistant 12-5 (the GM maize DR12-5) was used as a template in the experiments. The amplification results were detected using visual detection or fluorescent signal.

### 2.7. Sensitivity and Specificity of SMART 

The transgenic maize containing DR12-5 was used to determine the limits of detection (LOD) of the SMART. A dilution series of DR12-5 genomic DNAs at concentrations of 50, 100, 200, 300, 400, and 500 copies was prepared. The specificity of the SMART was evaluated using 12 samples (1 GM maize DR12-5 samples, 3 GM rapeseed MS1 × RF1 samples, 3 GM soybean 356043 samples, and 3 GM maize NK603 samples). Additionally, all the samples were tested by RPA and RT-PCR using the GM DR12-5 primers. 

### 2.8. Real Sample On-Site Detection Using SMART 

A total of 30 samples from plants (including 2 GM maize DR12-5 samples), animals (including 2 duck meat samples), and microbes (including 2 Ac samples) were used to evaluate the applicability of the SMART for on-site detection of real samples. These samples were named from M 1 to M 30. All the genomic DNA was adsorbed by silicon film and used as the template for both RT-PCR and SMART. 

## 3. Results and Discussions

### 3.1. DNA Adsorption System

#### 3.1.1. Performance Evaluation of DNA Adsorption Materials

**Figure 3:**
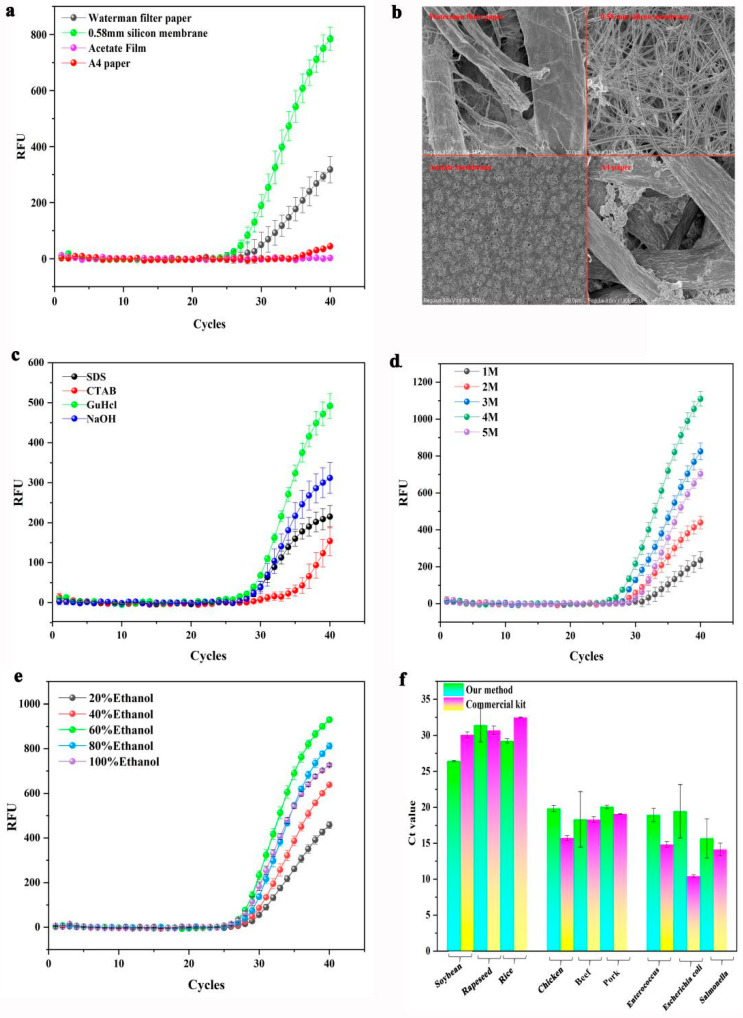
Establishment of ultrafast DNA adsorption system. Each experiment in this work was repeated three times independently: (**a**) selection of adsorption materials; (**b**) microscopic images of adsorption materials; (**c**) screening of lysis buffer; (**d**) the PCR amplification curves of amplicons from five concentrations of guanidine hydrochloride lysate adsorbed DNA as targets; (**e**) screening results of washing solution; (**f**) the PCR amplification results in different samples of nucleic acids adsorbed by SMART.

The results of the four materials are provided in Figure 3a. According to the RT-PCR results, 0.58 mm silicon film showed the strongest fluorescence result, followed by Waterman filter paper with a weak fluorescence result, A4 paper, and cellulose acetate paper with no fluorescence result. This trend may be related to the composition of the material. According to previous reports [18], the material surface of 0.58 mm silicon film included the Si element, fabricated by glass fiber composed of SiO_2_, which makes the silicon film surface positively charged. This is a key reason why 0.58 mm silicon film can easily adsorb DNA at high salt concentrations. These results aligned with previous reports. Waterman filter paper contains an abundance of cotton fibers and has a weak negative polarity, allowing it to readily adsorb DNA at a high salt concentration. Additionally, cellulose acetate paper is predominantly composed of cellulose acetate, which may possess a strong negative charge that prevents it from adsorbing DNA at high salt concentrations. A4 paper is mainly composed of plant fibers, which may not be charged, resulting in its inability to adsorb DNA at high salt concentrations.

Additionally, the fiber diameter and pore size of the four materials differed significantly. According to previous reports, the adsorption capacity of a material is closely related to its fiber diameter and pore size. Observing electron microscope images, the fiber diameter of these materials was A4 paper > Waterman filter paper > cellulose acetate paper > 0.58 mm silicon film (Figure 3b), indicating that A4 paper had the largest fiber diameter, while the 0.58 silicon film had the smallest. At the same magnification, the aperture of these materials was A4 paper > Waterman filter paper > 0.58 mm silicon film > cellulose acetate paper (Figure S4). These results indicated that an excessive fiber diameter might reduce the adsorption capacity of the material. Additionally, the adsorption capacity of the substance is affected by the pore size of the material. The moderate fiber and pore size of the 0.58 mm silicon film made its adsorption capacity greater than that of the other 3 materials. These results suggested that the 0.58 mm silicon film met the requirements of the adsorption material; thus, it was employed as an adsorbent.

#### 3.1.2. Performance Evaluation of Lysis Buffer 

The screening results of the lysis liquid are provided in Figure 3c. Comparing the amplification curves of the 4 lysis liquids, the GuHCl lysis buffer demonstrated the strongest fluorescence result, followed by the 10%CTAB and 20%SDS lysis buffers, which presented weak fluorescence results, and finally the NaOH lysis buffer, which showed the weakest fluorescence result. Additionally, the Tt values of these lysis buffers were: 1M-NaOH > 20%SDS > 10%CTAB > 4M-GuHCl. These findings validated the cleavage properties of the GuHCl lysis buffer, which was suitable for rapidly lysing various samples.

For optimal results, the concentrations of GuHCl lysis buffer were evaluated. In this work, 0.58 mm silicon film was used to adsorb DNA from non-GM seeds, which were then used as a template for RT-PCR amplification of the endogenous plant gene (18S rRNA). To determine the optimal concentration of the GuHCl lysis buffer, the DNA adsorption by 0.58 mm silicon film was performed at different lysis solution concentrations (1, 2, 3, 4, and 5 M). The detection results of RT-PCR for the GuHCl lysis buffer ranging from 1 to 5 M demonstrated that each reaction produced fluorescence values of varying intensities. According to the detection results of RT-PCR, the Tt value of these lysis buffers were 1 M > 5 M > 2 M > 3 M > 4 M. Their end point fluorescence value was 4 M > 3 M > 5 M > 2 M > 1 M (Figure 3d). These results indicated that the lysis performance of 4 M GuHCl was better than that of 3 M GuHCl, which was superior to that of 1, 2, and 5 M GuHCl. Comparing the lysis performance of these lysis buffers revealed the following potential causes for these results: lower concentration (1, 2, and 3 M GuHCl) of guanidine salt solution may reduce the DNA capture efficiency of silica film; additionally, its too high (5 M GuHCl) concentration denatured the enzymes, inhibiting enzyme activity in the PCR reaction. As a result, 4 M GuHCl lysis buffer was used for lysing.

#### 3.1.3. Washing Performance Evaluation of Washing Buffer

The screening result of the washing buffer is depicted in Figure 3e. Comparing the results in Figure 3e, there was almost no significant difference in the Tt value from 20 to 100% ethanol. Additionally, the end point fluorescence values of these washing liquids were as follows: 60 > 80 > 100 > 40 > 20% ethanol, indicating that the washing performance of 60% ethanol was better than 80 and 100% ethanol, which was superior to that of 20% and 40% ethanol. Analysis of possible reasons behind these results included: 20% ethanol content in the washing solution resulted in insufficient washing and affected the subsequent RT-PCR reaction. On the other hand, the ethanol concentration in the washing solution was 100%, which may inhibit the enzyme’s activity in the RT-PCR reaction. Therefore, 60% ethanol was used as a wash solution in this study.

#### 3.1.4. Evaluation of Rapid DNA Adsorption Method 

The RT-PCR detection results are shown in Figure 3f. Using the commercial kit, the average Ct values for DNA from plants, animals, and microorganisms were 30.5, 16.8, and 13.2, respectively, while by using SMART, the mean Ct values of DNA from plants, animals, and microorganisms were 29, 19.5, and 18, respectively. 

Simultaneously, observing the amplification curve of amplicons (Figure S5) revealed that the fluorescence values of DNA adsorbed by SMART were comparable to those extracted using the commercial kit. These results illustrated that the SMART had comparable extraction performance to commercial kits and could effectively adsorb the DNA from plants, animals, and microbes. 

### 3.2. Temperature Control Experiments

**Figure 4:**
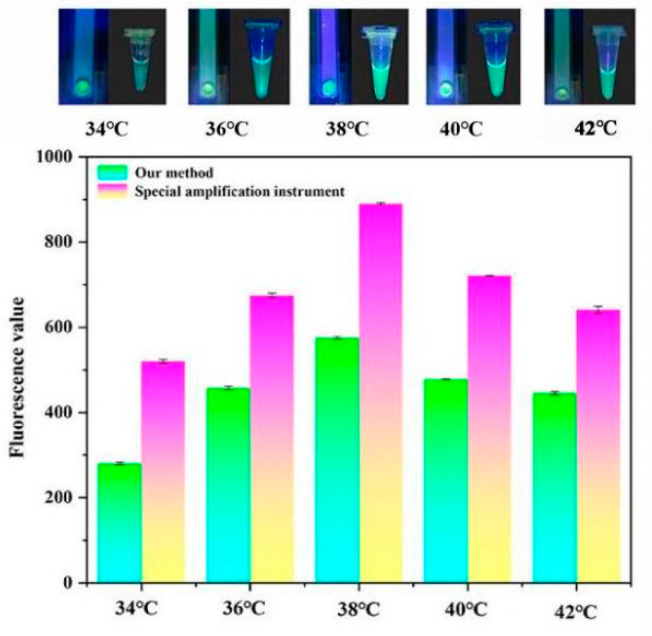
Evaluation of SMART temperature control performance. Visual detection results of the SMART (**top panel**); end point fluorescence value of visual detection results of target genes (**bottom panel**).

To evaluate the temperature control performance of SMART, we conducted the RPA reactions at different temperatures. In the SMART, reaction temperatures ranging from 34 to 42 °C generated green fluorescence with varying intensities for each reaction, with 34–38 °C green fluorescence increased gradually. The strongest fluorescence was observed at 38 °C. The green fluorescence weakened gradually at higher temperatures (40–42 °C). These results were consistent with those obtained using a thermal block (Figure 4 top panel). 

To further assess the temperature control performance of SMART, a microplate scanning spectrophotometer (BioTek, Shoreline, WA, USA) was utilized to determine end point fluorescence values of two heating methods at varying temperatures. These values showed that the end point fluorescence value gradually increased from 34–38 °C and reached its maximum at 38 °C (Figure 4 bottom panel). At 40–42 °C, the end point fluorescence value gradually decreased as the temperature increased. These results were consistent with the preceding ones. These findings suggested that SMART has efficient temperature control performance.

### 3.3. Sensitivity and Specificity of SMART

**Figure 5:**
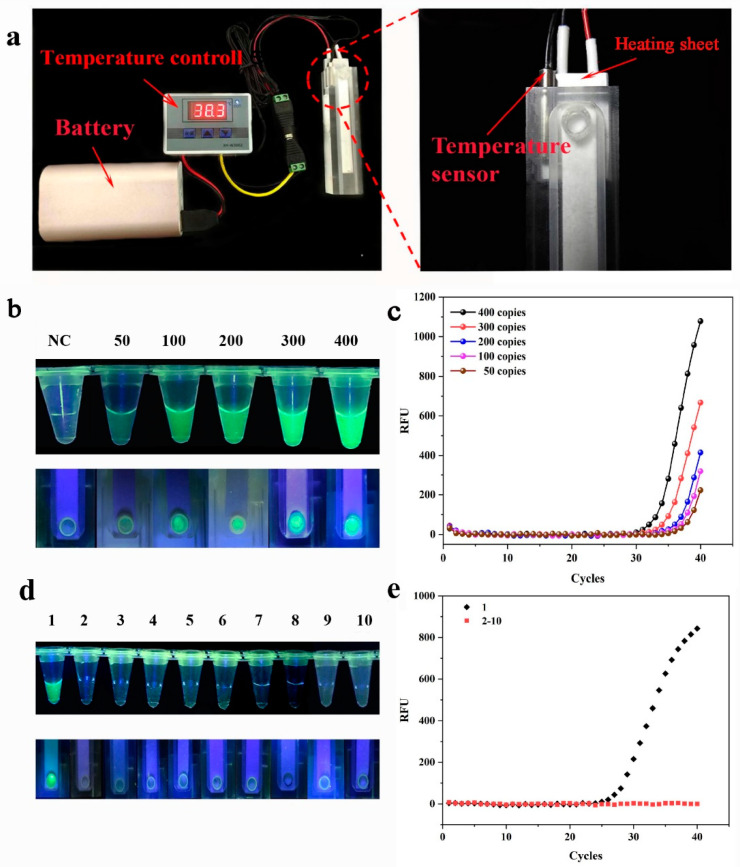
Sensitivity and specificity of SMART: (**a**) the photo of the SMART amplification module; the image on the right is of the temperature probe and the heating plate; (**b**) sensitivity performance of SMART and RPA with different copy concentrations of genomic DNA; visual detection results of RPA in the laboratory (top panel); visual detection results of SMART (bottom panel); (**c**) sensitivity of the GM DR12-5 using RT- PCR; (**d**) the visual detection results of specificity; the visual detection results of the RPA in the laboratory (top panel); visual detection results of SMART (bottom panel); 1: the GM maize DR12-5; 2, 3, 4: the GM rapeseed MS1×RF1; 5, 6, 7: the GM soybean 356043; 8, 9, 10: the GM corn NK603; (**e**) the specificity of the different sample DNA was detected using RT-PCR.

The SMART amplification module is shown in Figure 5a. The LOD of the SMART was evaluated by testing several dilutions of DR12-5 at concentrations of 50, 100, 200, 300, and 400 copies per reaction. The results suggested that the LOD for DR12-5 was 50 copies/reaction. A comparable LOD was obtained with the RPA reaction in the tube (Figure 5b). Additionally, analyzing the PCR amplification curve revealed that the LOD of PCR was identical to that of SMART (Figure 5c). At the same time, the end point fluorescence values of the SMART and RPA reactions in the tube were analyzed, and it was realized that they had comparable fluorescence values (Figure S6). These results indicate that the LOD of SMART was 50 copies/reaction.

The specificity of the SMART assay was verified using 12 samples of plants. In this work, only the GM maize DR12-5 sample exhibited fluorescence, whereas no fluorescence was observed in other plant varieties. As expected, the detection results of the SMART were consistent with the RPA reaction in the laboratory (Figure 5d). Simultaneously, RT-PCR was used to detect the same samples, and the results were consistent with those obtained using SMART. This indicated that the detection method established in our study has a high degree of specificity.

### 3.4. On-Site Detection Results of SMART 

**Figure 6:**
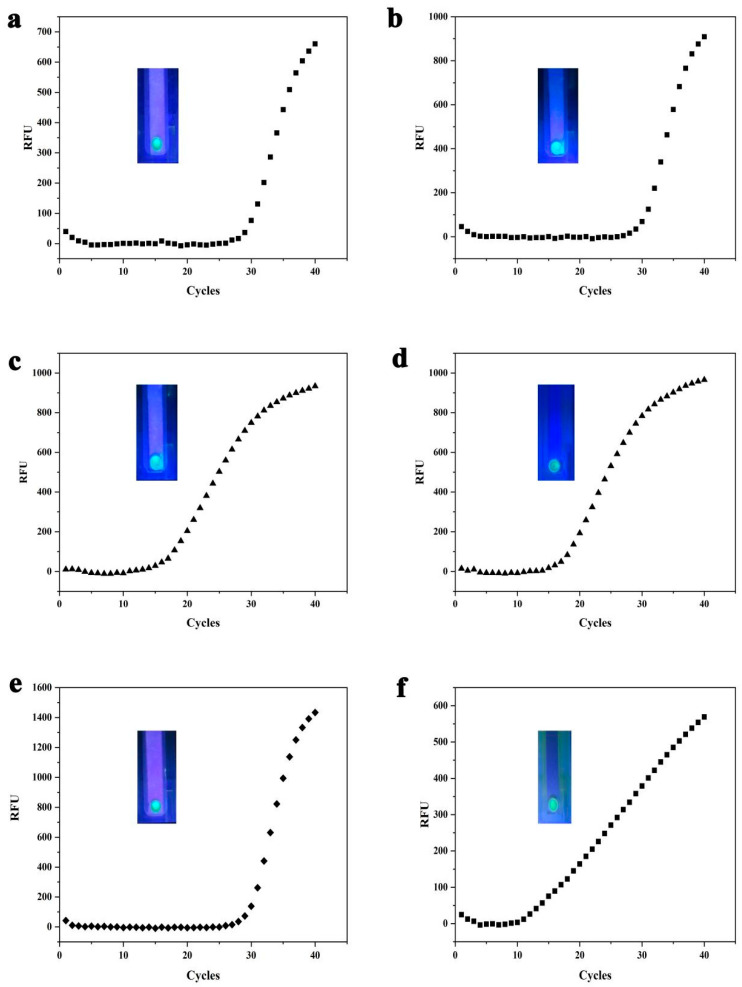
On-site detection results of SMART: (**a**) test results of M2 samples (the GM maize DR12-5); (**b**) test results of M5 samples (the GM maize DR12-5); (**c**) test results of M7 (duck meat); (**d**) test results of M9 (duck meat); (**e**) the test results for M21 (Ac); (**f**) the test results for M28 (Ac).

Using the developed system, DNA was successfully detected in 6 samples (two GM maize DR12-5, 2 duck meat, and 2 Ac samples) during on-site testing. In all the 30 tested samples (M1-M30), M2 and M5 were GM maize DR12-5 (Figure 6a,b), M7 and M9 were duck meat (Figure 6c,d), M21 and M28 were Ac samples (Figure 6e,f). The test results of SMART were consistent with RT-PCR’s detection results (Figure 6). We compared the previously reported detection methods (Table 1). Unlike traditional PCR detection methods, SMART does not use expensive thermal chermal circulation systems. Additionally, our detection method is simple, fast, comparable to protein test strips, and more widely used. The SMART is highly integrated with ultrafast DNA adsorption, target gene amplification, and visual detection, with the detection of amplicons in less than 25 min. Additionally, SMART showed good temperature control performance, which accurately regulated the temperature of the RPA reaction. Therefore, our method proved superior to the current RPA method (event-specific RPA).

## 4. Conclusions

This study developed SMART, a highly integrated technology with ultra-fast DNA adsorption, rapid amplification of target genes, and a visual detection device capable of detecting DNA from plants, animals, and microbes. With optimized adsorption material, lysis buffer, and washing buffer, the process, from ultra-fast DNA adsorption to result detection, can be completed in 25 min. Rapid detection of genetically modified maize using SMART showed high sensitivity and selectivity. The LOD for detecting transgenic maize genomic DNA with SMART can exceed 50 copies. SMART was used to detect real samples from plants, animals, and microbes, and the results were consistent with conventional PCR, indicating that SMART is more robust. Additionally, SMART is low-cost and requires no specialized heating equipment, making it suited for usage in limited resource areas.

## Figures and Tables

**Table 1:** Comparison of different detection methods.

Analytical Method	Target	^a^ Instrument	^b^ Time for Detection	Sensitivity	Suitability for Controlling the Temperature	Suitability for On-Site-Testing	Ref.
ELISA	Protein	Need	>5 h	0.25%	Unsuitable	Unsuitable	[17]
Protein strip test	Protein	No need	3–5 min	0.1%	Unsuitable	Suitable	[19]
Conventional PCR	DNA	Need	>3 h	8 copies	Suitable	Unsuitable	[20]
RT-PCR	DNA	Need	>2.5 h	1 copy	Suitable	Unsuitable	[21]
DRPA-LFB	DNA	No need	About 30 min	10 copies	Unsuitable	Suitable	[16]
Event-specific RPA	DNA	No need	25 min	0.1%	Unsuitable	Suitable	[15]
Our method	DNA	No need	≤25 min	50 copies	Suitable	Suitable	This study

^a^ It refers to the professional equipment or instrument required for the detection process; ^b^ it refers to the entire detection time from the extraction of protein or DNA to the final determination of the target.

## Data Availability

The data presented in this study are available on request from the corresponding author.

## Supplementary Materials

The following supporting information can be downloaded at: https://www.mdpi.com/article/10.3390/bios13010082/s1, Figure S1: A schematic of silicon membrane adsorbing DNA; Figure S2: Schematic of SMART detailed operation; Figure S3: The image of a disposable punch; Figure S4: The fiber diameter of four paper-based nucleic acid adsorption materials; Figure S5: The amplification curve of the standard sample. Each experiment in this study was repeated three times independently. (a) RT-PCR amplification of the 18S rRNA gene from plant samples DNA. (b) RT-PCR amplification of the 18S rRNA gene from animal samples DNA. (c) RT-PCR amplification of the 16S rRNA gene from microorganism samples DNA; Figure S6: The end-point fluorescence values of different genomic DNA copy numbers; Table S1: Primers and probes used in this study. References [22,23] are cited in Supplementary Materials.
