# Supplementary material for: SMART: On-Site Rapid Detection of Nucleic Acid from Plants, Animals, and Microorganisms in under 25 Minutes
Source: Biosensors (Basel). 2023 Jan 3;13(1):82. doi: 10.3390/bios13010082 (PMC9855345; doi:10.3390/bios13010082)
Supplement: Supplementary file 1 [file biosensors-13-00082-s001.zip › biosensors-2108758-supplementary.pdf]

Supplementary information for

SMART: On-Site Rapid Detection of Nucleic Acid from Plants, Animals, and Microorganisms in under 25 Minutes

Jun-Yuan Ma [1,2], Xiao-Fu Wang [1,3,*], Cheng Peng [1,3], Xiao-Yun Chen [1,3], Xiao-Li Xu [1,3], Wei Wei [1], Lei Yang [1], Jian Cai [2] and Jun-Feng Xu [1,3,*]

1 State Key Laboratory for Managing Biotic and Chemical Threats to the Quality and Safety of Agro-Products, Zhejiang Academy of Agricultural Sciences, Hangzhou 310021, China
2 College of Biological and Food Engineering, Fuyang Normal University, Fuyang 236041, China
3 Key Laboratory of Traceability for Agricultural Genetically Modified Organisms, Ministry of Agriculture and Rural Affairs, Hangzhou 310021, China
* Correspondence: yywxf1981@163.com (X.-F.W.); njjfxu@163.com (J.-F.X.).

SI-1 The *principle of silicon membrane adsorbing DNA*

According to previous reports [1], high concentrations of salt solution contribute to the nucleic acid adsorption efficiency of silicon-based materials. Here, we are based on this principle to ultrafast adsorb DNA from samples. When the DNA sample solution is loaded into the bottom of the silicon film（labeled with "◯"）, the sample solution moistens the silicon membrane and then DNA and impurities are separated through capillary force. Next, DNA is adsorbed at the bottom of the silicon membrane and impurities are migrated to the top of the silicon membrane.

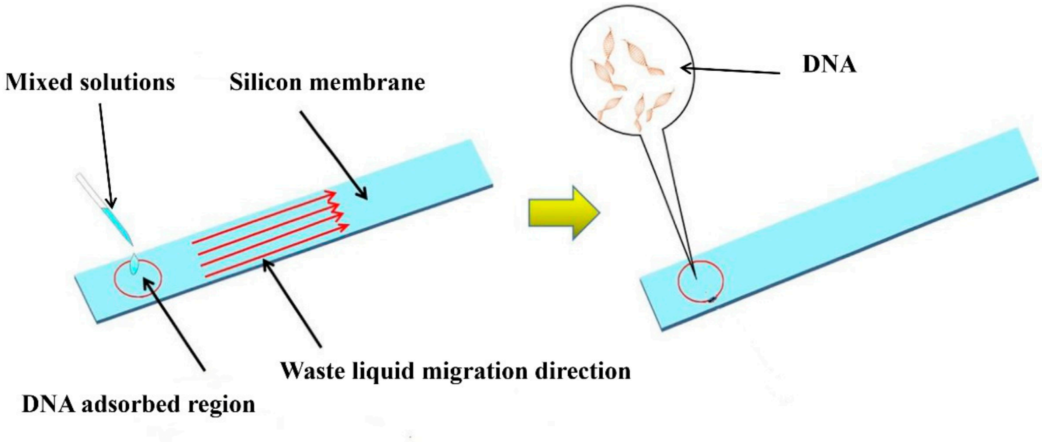

Figure S1. A schematic of silicon membrane adsorbing DNA.

SI-2 *The detailed operating process of SMART*

After ultrafast adsorption of DNA from a sample using SMART, 200 μL of lysis liquid containing 4 M GuHCl were put into a 1.5 ml tube that contains the sample. The mixture was shaken vigorously by hand for 1min, and it was then well mixed. Later the 60 μL homogenized mixture was transferred to the bottom of the silicon film in SMART, and the upper plate was closed, incubate for 2 min. Then open the upper plate again, about 200 μL wash liquid containing 60% ethanol was injected into the silicon film on the SMART, incubate for 2 min. Next, the silicon film containing DNA is cut by pressing the silicon film cutter. Then, 50ul RPA reaction solution was added to the silicon film from the top of the silicon film cutter. In addition, use polyethylene sealing film to quickly seal the top of the silicon film cutter. The heating plate and temperature

sensor were inserted into the rectangular groove and hole on the side of SMART, respectively. The power(5V) was switched on to amplify the target DNA.The RPA reaction was incubated at 38 ˚C for 20 min. In this work, we designed an Exo probe, which introduced a base mimic tetrahydrofuran (THF) modification site inside the probe, and coupled fluorescent groups and quenching groups on both sides near the site. When the probe hybridizes with the target sequence on the silicon membrane to form double-stranded DNA, Exo recognizes the THF modification site and cuts the position so that the fluorescent group and the quenching group are separated, thereby generating fluorescence [2]. When there is no target on the silicon film, Exo cannot recognize the THF site, which separates the fluorescent group from the quenching group and thus cannot produce fluorescence. After completing the above operation, The visual green fluorescence result for product detection was obtained with glasses under a hand-held lamp (LUYOR-3415RG).

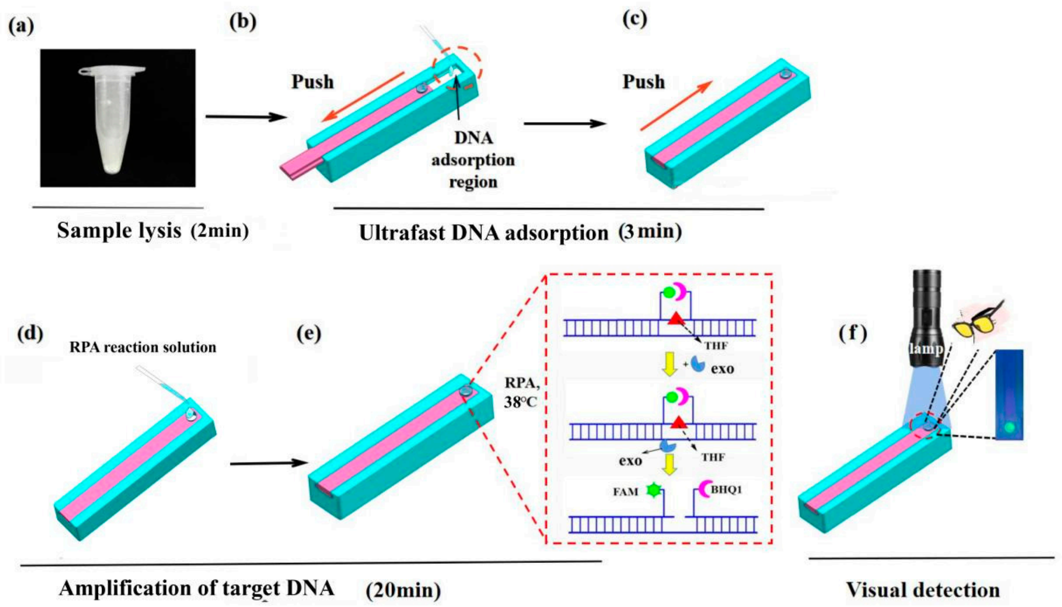

Figure S2. Schematic of SMART detailed operation.

SI-3 ***The disposable punch structure and use instructions***

The disposable punch consists of three parts, including the punch, spring, and empty cylinder. The procedure for the disposable punch is as follows: insert the tip at the bottom of the disposable punch into the silicon film that had been adsorbed DNA. Then disposable punch the bottom of the tip put it into the PCR reaction tube and press the punch top to release the silicon particles. The PCR reaction tube was added about 18 μLPCR reaction solution for PCR reaction, via the RT-PCR system.

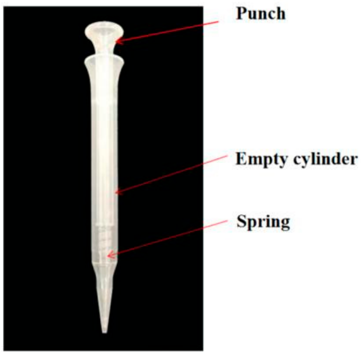

Figure S3. The image of a disposable punch.

SI-4 *Primers and probes in this study*

Primers and probes using RT- PCR, and RPA were synthesized according to the previous study. All primers and probes were synthesized by Sangon Biotech Co., Ltd. (Shanghai, China). Detailed information on all primers and probes is provided in Table S1.

Table S1. Primers and probes used in this study

Purpose	Name	Sequence (5' - 3')
RPA for GM-DR12-5	RPA-12-5-F	GACGTTCGGTGCCTGGAAGACAAGTTCTAC
	RPA-12-5-R	TGAATGGCGAATGCTAGAGCAGCTTGAGCT
	RPA-12-5-P	AACCACATCGCCCGACGCTACAACGA-GAC[FAM-dT][THF]A[BHQ-dT]AGTTTAAACTGAA[3'-block]
RPA for duck	duck-F	GGAGCACCTCTATCAGAGAAAGACA
	duck-R	GTGTGTAGAGCTCAAGATCAATCCC
	duck-P	FAM-TGGGAACAAGCATGAATGTAAGTGGATGGT-BHQ1
RPA for Ac	Ac-F	GTTTTGACGCAATCAAATTTTTGTCACCGG
	Ac-R	CGGCTTCGCGAGAGGCCTCTTTGTTGTTGG
	Ac-P	TTCCGCCGGCAACGCTGATTCGACTC-TA/i6FAMdT/G/idS/A/iBHO1dT/TTTTAAAGAACAG
RT-PCR for18S	18S-F	AGCTCGTAGTTGGATTTCTGTTAATAATTTA
	18S-R	TATGCCTGCTTTAAGCACTCT
	18S-P	FAM-TTTCTCAAAGTAAAATTTCA-BHQ1
RT-PCR for16S	16S-F	TCCTACGGGAGGCAGCAGT
	16S-R	GGACTACCAGGGTATCTAATCCTGTT
	16S-P	FAM-CGTATTACCGCGGCTGCTGGCACBHQ1
RT-PCR for duck	duck-F	GGAGCACCTCTATCAGAGAAAGACA
	duck-R	GTGTGTAGAGCTCAAGATCAATCCC
	duck-P	FAM-TGGGAACAAGCATGAATGTAAGTGGATGGT-BHQ1
RT-PCR for Ac	Ac-F	CTGATAATCCTCGGCTCCAACAA
	Ac-R	TGAGCGCATTTCTGACGAG
	Ac-P	FAM-AAGAAATACGCCCTCGCCAATCTCC-BHQ1
RT-PCR for GM-DR-12-5	12-5-F	GTCGTTTCCCGCCTTCAGTT
	12-5-R	GGTGCCTGGAAGACAAGTTCTA
	12-5-P	FAM-AGCTCAACCACATCGCCCGACGC-BHQ1

SI-5 *Fiber diameter of four DNA adsorption materials*

The fiber diameters of Waterman filter paper, 0.58 mm silicon membrane, cellulose acetate paper, and A4 paper were observed by scanning an electron microscope at the same magnification.

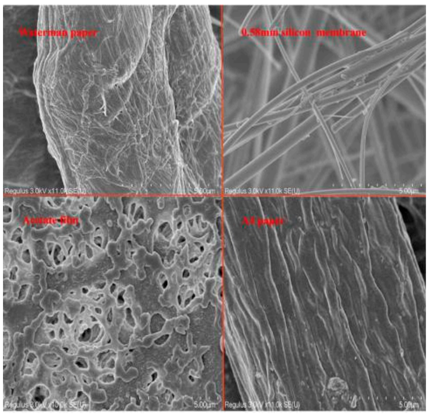

Figure S4. The fiber diameter of four paper-based nucleic acid adsorption materials.

SI-6 *Rapid adsorption of DNA using SMART from different samples*

To evaluate the feasibility of this novel method for rapid DNA adsorption, samples from plants, animals, and microorganisms were used for DNA extraction by SMART and commercial kits.

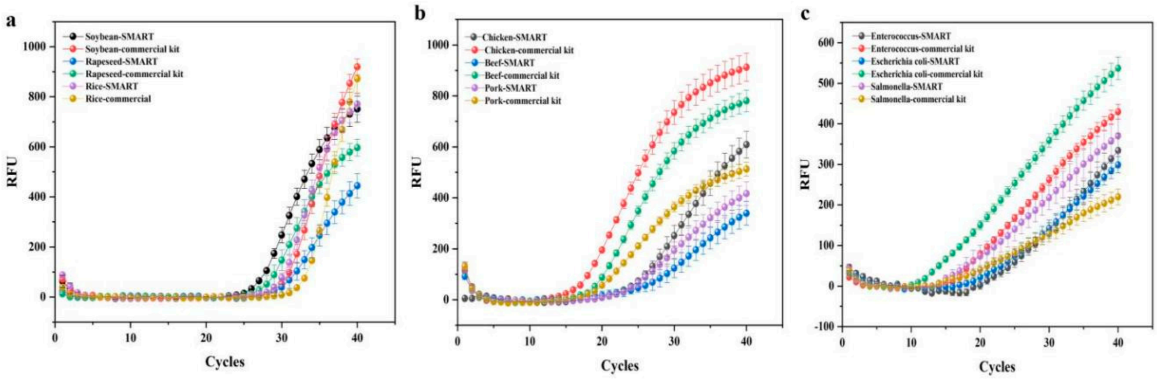

Figure S5. The amplification curve of the standard sample. Each experiment in this study was repeated three times independently. **(a)** RT-PCR amplification of the 18S rRNA gene from plant samples DNA. **(b)** RT-PCR amplification of the 18S rRNA gene from animal samples DNA. **(c)** RT-PCR amplification of the 16S rRNA gene from microorganism samples DNA.

SI-7 *Endpoint fluorescence detection under different genomic DNA copy numbers*

The end-point fluorescence values of two different RPA reactions at different copy numbers were measured, using a microplate scanning spectrophotometer.

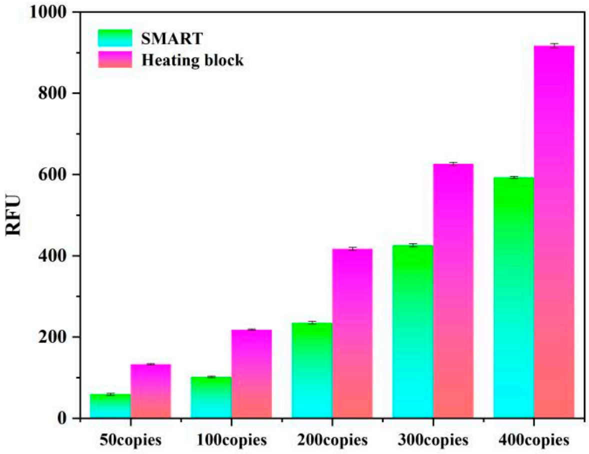

Figure S6. The end-point fluorescence values of different genomic DNA copy numbers.

References

1.Mason; M.G.; Botella; J.R.; Nat. Protoc. **2020,** 15, 3663 – 3677. https://doi.org/10.1038/s41596-020-0392-7.

2. Wang Z; Wang Y; Lin L; et al. A finger-driven disposable micro-platform based on isothermal amplification for the application of multiplexed and point-of-care diagnosis of tuberculosis. Biosensors and Bioelectronics. **2022**, 195, 113663. https://doi.org/10.1016/j.bios.2021.113663.
